# Exopolysaccharides from the Energy Microalga Strain *Botryococcus* *braunii*: Purification, Characterization, and Antioxidant Activity

**DOI:** 10.3390/foods11010110

**Published:** 2022-01-01

**Authors:** Wei-Nan Wang, Tao Li, Yi Li, Ying Zhang, Hua-Lian Wu, Wen-Zhou Xiang, Ai-Fen Li

**Affiliations:** 1 CAS Key Laboratory of Tropical Marine Bio-resources and Ecology, Guangdong Key Laboratory of Marine Materia Medica, Institution of South China Sea Ecology and Environmental Engineering, South China Sea Institute of Oceanology, Chinese Academy of Sciences, Guangzhou 510301, China; wangweinan0220@163.com (W.-N.W.); taoli@scsio.ac.cn (T.L.); hlwu@scsio.ac.cn (H.-L.W.); 2 University of Chinese Academy of Sciences, Beijing 100049, China; 3 Southern Marine Science and Engineering Guangdong Laboratory (Guangzhou), Guangzhou 511458, China; 4 Engineering Research Center for Tropical and Subtropical Aquatic Ecological Engineering, Ministry of Education, Jinan University, Guangzhou 510632, China; liyi1biology@163.com (Y.L.); zhangying7005@163.com (Y.Z.)

**Keywords:** *Botryococcus braunii*, L race, exopolysaccharides, chemical composition, antioxidant activity

## Abstract

*Botryococcus* *braunii*, a prestigious energy microalga, has recently received widespread attention because it can secrete large amounts of exopolysaccharides (EPS) with potential applications in food, cosmetics, and nutraceuticals. Unfortunately, the insufficiency of research on the bioactivity and structure–activity relationship of *B*. *braunii* EPS has impeded the downstream applications. In the present study, alcohol precipitation, deproteinization, and DEAE-cellulose column chromatography were used to extract and purify *B*. *braunii* SCS-1905 EPS. It was found that *B*. *braunii* SCS-1905 EPS were high-molecular-weight heteropolysaccharides containing uronic acid (7.43–8.83%), protein (2.30–4.04%), and sulfate groups (1.52–1.95%). Additionally, the EPS primarily comprised galactose (52.34–54.12%), glucose (34.60–35.53%), arabinose (9.41–10.32%), and minor amounts of fucose (1.80–1.99%), with the presence of a pyranose ring linked by a *β*-configurational glycosidic bond. Notably, the antioxidant activity of crude exopolysaccharides (CEPS) was stronger, and the half maximal inhibitory concentration (IC_50_) for ABTS and hydroxyl radicals was significantly lower than that of deproteinized exopolysaccharides (DEPS). Overall, this study indicated a potential application of *B*. *braunii* SCS-1905 EPS as a natural antioxidant. In summary, *B*. *braunii* EPS could be used as a potential feedstock for the production of antioxidant health foods.

## 1. Introduction

Exopolysaccharides (EPS), known as extracellular polymeric substances, are macromolecular mixtures of polysaccharides secreted by microalgae into the extracellular matrix during the life cycle [1]. Exopolysaccharides are ecologically essential, besides protecting cells from dehydration and toxic substances and acting as energy and carbon sinks in response to nutrient stresses [2]. They also contribute to promoting the formation of algal aggregates, mediate cell-matrix adhesion and play an important role in the stability of biofilm structure [3].

The potential for EPS to be used in a variety of industries, including food, medicine, cosmetics, manufacturing, etc. has made it economically appealing and is increasingly becoming a research hotspot for scientists. Microalgal EPS were first developed as thickeners and bio-lubricants in food products due to their unique physical properties such as high viscosity and excellent rheological properties [4,5]. The chemical composition and structural properties of polysaccharides can significantly affect the physicochemical properties and biological activities. Studies on the application of EPS are gradually diversifying as a growing number of species of microalgal EPS are identified. EPS, like biomass, has a high value for food production as a carbon pool. Microalgal polysaccharides or their derivatives contain soluble dietary fibers, which are not fermented, at least not completely, by colonic microbiota, can positively regulate the microbiota associated with obesity and diabetes in the human gut, playing a major role in human health and diseases [6].

Numerous studies have suggested that microalgal EPS possess various biological activities such as antioxidant, anti-inflammatory, antiviral, and antitumor properties, which is expected to be a potential feedstock for nutritional supplements, cosmetics, and pharmaceuticals. As by-products of the microalgae culture process, EPS can be dissolved in the culture medium, that makes the preparation process simpler compared to intracellular products and has more advantages for industrialization. For instance, *Porphyridium cruentum* EPS have antioxidant, antibacterial and antiviral properties; the carrageenan induced paw edema test revealed that EPS from *Chlorella stigmatophora* and *Phaeodactylum tricornutum* possess an anti-inflammatory ability; *Aphanothece halophytica* EPS (EPAH) was proven effective in the treatment of pneumonia caused by influenza virus A FM (H1N1) [7,8,9,10]. At present, microalgal EPS has been believed to have broad application prospects. Frutarom Industries Ltd. (Israel) has successfully extracted the EPS and converted it into cosmetics with large-scale cultivation. Admittedly, no other species of microalgal EPS have achieved commercial applications.

The unicellular colonial microalga *B*. *braunii* is a member of the Trebouxiophyceae (Chlorophyta) widespread in fresh and brackish waters of temperate and tropical zones [11]. *B*. *braunii* has been widely regarded as a promising source of sustainable bioenergy production due to its capability to produce large amounts of lipids and hydrocarbons (up to 86% of dry weight). According to the characteristic hydrocarbon they produce, *B*. *braunii* have been classified into four chemical races A, B, L, and S. Race A accumulates odd-numbered unbranched alkadienes and trienes (C23–C33); race B accumulates polymethylated triterpenes C_n_H_2n10_ (C30–C37), the botryococcenes [12]; race L is characterized by single tetraterpenoid hydrocarbon called lycopadiene [13], and race S produces hydrocarbon comprising epoxy-n-alkane and saturated n-alkane chains with carbon numbers 18 and 20 [14].

In addition to its excellent performance in the production of hydrocarbons and lipids, *B*. *braunii* can secrete large amounts of EPS, making it promising for commercial production and applications. Casadevall et al., (1985) observed a large amount of glycofibrils attached to algal cells through transmission electron microscopy for the first time [15]. A non-axenic strain of the microalga *B*. *braunii* UC 58, isolated from a small lake in Portugal by Fernandes et al., (1989), showed remarkably high quantities of EPS (up to 4.5 g L^−1^), which contained fucose and galactose in a proportion of 0.6: 1 [16]. Allard and Casadevall (1990) studied the EPS of five strains of *B*. *braunii* from three different chemical races A, B, and L. Among them, the EPS production of A and B strains was about 0.25 g L^−1^, while the production of the L strain was up to 1.0 g L^−1^. Galactose accounted for the main part of *B*. *braunii* EPS [17]. So far, there have been large amounts of studies on influencing factors of EPS production and outdoor cultivation of *B*. *braunii*. However, there are few studies of the biological activity of *B*. *braunii* EPS. The biological activity of EPS deserves our attention and is an important theoretical foundation for commercial applications in the future.

In this study, the microalgae strain *B*. *braunii* SCS-1905 was newly isolated from a small lake in Guangdong Province, China, by the authors of this study. During the cultivation period, a large amount of uplifted foam and viscous texture appeared in the culture medium. After extraction, isolation, and purification of the medium, several different EPSs were obtained, including CEPS (crude exopolysaccharides), DEPS (deproteinized exopolysaccharides), and purified fractions of EPS-1, EPS-2, and EPS-3, and described in terms of chemistry and primary structure. In addition, antioxidant activities in vitro of the EPS were investigated to evaluate the commercial application potential as well as to explore the potential development and utilization.

## 2. Materials and Methods

### 2.1. Microalgae and Culture Conditions

*B*. *braunii* SCS-1905, isolated from a small lake on Panyu District in Guangzhou (Guangdong Province, China), was preserved in South China Sea Institute of Oceanology, Chinese Academy of Sciences (Guangzhou, China). It was inoculated in a glass column photobioreactor (Ø6.0 cm × 60 cm) containing modified BG-11 medium (mBG-11) [18]. The culture period was 20 days. During this period, the strain was bubbled with CO_2_-enriched compressed air (1% CO_2_, *v*:*v*), filtered through a 0.2 μm sterile disposable filter were provided with 200 μmol photons·m^−2^ s^−1^ one-side illumination by T8 fluorescent lamp (Philips, Eindhoven, The Netherlands) at 25 ± 1 °C.

### 2.2. Morphological Observation and Phylogenetic Tree Construction

Morphological characteristics of the cells were observed under an Olympus BX41 microscope (Olympus Corporation, Tokyo, Japan). The CTAB (conventional cetyltrimethylammonium bromide) method was used for DNA extraction as described by Chen et al., (2014) [19]. A (5′-CTGGTTGATCCTGCCAGT-3′) and B (5′-CACCTACGCAAACCTTGTTACGACTT-3′) were used as primers for the 18S rRNA gene sequence. The PCR reaction was carried out using the KOD–Plus–Neo DNA polymerase (Toyobo, Osaka, Japan) according to the procedure described by Li et al., (2016) [18]. The PCR procedure include that: (1) pre-denaturation at 94 °C for 3 min, (2) denaturation at 94 °C for 15 s, (3) annealing at 5 °C for 30 s, (4) extension at 68 °C for 7 min, (5) the process was repeated for 30 cycles. DNA gel extraction kit (TIANGEN Biotech., Beijing, China) was used to recover and purify PCR results. The purified PCR result was sequenced. The phylogenetic tree was built using ClustalX 1.8 and MEGA 4.0 software.

### 2.3. Preparation of Crude Exopolysaccharides (CEPS) and Deproteinized Exopolysaccharides (DEPS)

The algae cells were separated by centrifugation at 3996× *g* for 5 min, and the supernatant obtained was concentrated by a RE-2000A rotary evaporator (Yarong, Shanghai, China) at 50 °C. Subsequently, the concentrated supernatant was dialyzed against distilled water to remove small molecules (1 kDa cutoff). Finally, 95% ethanol was added to the supernatant (95% ethanol: supernatant = 4: 1, *v*:*v*). The collected flocs were crude exopolysaccharides (CEPS).

The Sevag method was used to remove protein from CEPS [20]. Briefly, 3 mL CEPS solution and 1 mL of the chloroform: n-butanol solution (4: 1, *v*:*v*) were added in a tube. After shaking vigorously for 30 min, the aqueous phase and the organic phase were separated by centrifugation. Polysaccharides exist in the aqueous phase while denatured proteins exist in the organic phase. The polysaccharide solutions collected after each deproteinization were scanned for the full-wavelength spectrum using the UV (ultraviolet)-visible spectrophotometer, and the absorbance at 280 nm was used as an indicator to determine the protein removal. The above operation was repeated 4–5 times, and then after alcohol precipitation and lyophilization, deproteinized exopolysaccharides (DEPS) were collected.

### 2.4. Purification by Ion Exchange Chromatography of Exopolysaccharides (EPS)

A cellulose DE-52 column (Ø26 mm × 0.3 m, 50μm) (newprobe, Beijing, China) equilibrated with distilled water was used to fractionate the DEPS solution in distilled water. The DEPS was prepared as a 10 mg mL^−1^ polysaccharide solution that was added to the ion exchange column wall after it had been pre-equilibrated with distilled water. The column was eluted with distilled water, followed by 0.4, 0.8, and 2.0 mol L^−1^ NaCl solution at 1 mL min^−1^ in order. The elution volume of each phase was 100, 200, 200, and 100 mL, respectively. In each tube, 10 mL aliquots were collected, and the carbohydrate content was determined using the phenol sulfuric acid method [21].

### 2.5. Determination of EPS Composition

Total carbohydrate content was determined by the phenol sulfuric acid method [20]. Protein content was determined by the Lowry method (Folin-phenol method), which is based on the biuret reaction by Oliver H. Lowry [21]. The peptide bond in the protein reacts with Cu^2+^ under alkaline conditions to form a protein–Cu^2+^ complex. Folin-phenol reagent can be reduced by this complex, and there is a linear relationship between the intensity of the blue color (750 nm) and the protein content. The uronic acid content was measured using a colorimetric method based on the meta-hydroxyphenyl reagent [22]. The sulfate content was calculated as reported by Reim (1991) [23]. With 2 mL of 1 mol L^−1^ hydrochloric acid, the EPS was hydrolyzed for 6 h at 100 °C. After filtering with a 0.45 μm microporous membrane, the distilled water volume was increased to 5 mL, and the sulfate content was measured using a DIONEX ICS-2500 ion chromatography (DIONEX, Sunnyvale, CA, USA).

The EPS and reference samples were derivatized according to the procedure described by Luo et al., (2010) [24]. The sample (5 mg) was dissolved in 2 mL of 2 M trifluoroacetic acid solution to hydrolyze at 110 °C for 2 h, and then repeatedly co-distilled with methanol to dryness. Hydroxylamine hydrochloride, inositol and pyridine were added to the sample hydrolysate and shaken at 90 °C for 30 min. Subsequently, acetic anhydride was added and shaking at 90 °C for 30 min. After cooling, the aldonitrile acetate derivatives were obtained to analyze the monosaccharide composition of EPS. The monosaccharide components were determined by GC-2014 gas chromatograph (Shimadzu, Kyoto, Japan) equipped with a flame ionization detector and an SH-Rtx-5 capillary column (30 m × 0.25 mm × 0.25 μm, Shimadzu, Kyoto, Japan). Argon was used as the carrier gas at a flow rate of 1 mL min^−1^. The temperature of the column was set to rise from 120 °C (with 3 min hold) to 210 °C in 3 min (with 4 min hold). The injection port and detector had temperatures of 250 °C and 280 °C., respectively. The injection volume was 1.0 L, with a 30: 1 split ratio.

### 2.6. Fourier Transform Infrared Spectroscopy (FT-IR) Analysis

KBr (200 mg) was added to the samples and KBr pellets were prepared for FT-IR analysis. The infrared spectrum of EPS (400–4000 cm^−1^) was examined with an IR affinity-1 Fourier transform infrared spectrometer (Shimadzu, Kyoto, Japan).

### 2.7. In Vitro Antioxidant Activity Assay

#### 2.7.1. ABTS (2,2′-Azinobis (3-Ethylbenzothiazoline-6-sulfonic Acid)) Radical-Scavenging Ability

The scavenging ability of EPS and DEPS against 2,2′-Azinobis (3-ethylbenzothiazoline-6-sulfonic acid) (ABTS) radicals was determined using the method described by Li et al., (2012) [25]. We mixed 7.4 mM ABTS diammonium salt and 2.6 mM potassium persulfate (K_2_S_2_O_8_) in an equal volume and left in the dark for 12 h. The mixture was diluted with phosphate buffer at pH 7.4 to achieve an absorbance of 0.70 ± 0.02 at 734 nm. 0.8 mL ABTS free radical working solution was added to 0.2 mL of samples at 1.0, 2.0, 3.0, 4.0, 5.0 mg L^−1^ concentrations, shaken and incubated at 37 °C for 15 min. Ascorbic acid was used as a positive control. The absorbance was determined at 734 nm at an Epoch™ 2 Microplate Spectrophotometer (Bio-Tek Instruments, Winooski, USA). The following equation was used to calculate the ABTS radical-scavenging activity:

Scavenging capacity (%) = (OD_0_ − OD_1_)/OD_0_ × 100 (1)

where OD_0_ represents the absorbance of the control group (distilled water rather than the sample solution), and OD_1_ represents the absorbance of the experimental group.

#### 2.7.2. Hydroxyl Radical-Scavenging Ability

Hydroxyl radical (·OH) scavenging activity was carried out according to Li’s methods (2008) with minor modification [26]. Reaction mixture was prepared by addition of 0.1 mL of each reagent including 0.75 mM 1,10-phenanthroline, 0.75 mM FeSO_4_, and 0.15 M phosphate buffer (pH 7.4). 0.1 mL of sample at various concentrations (1.0, 2.0, 3.0, 4.0, 5.0 mg L^−1^) was added to the reaction mixture, with 0.01% of H_2_O_2_ added in the end. Ascorbic acid was applied as a positive control. The absorbance was measured at 536 nm after 30 min of shaking at 37 °C. The following equation was used to calculate the hydroxyl radical-scavenging activity:

Scavenging capacity (%) = ((OD_1_ − OD_0_) / (OD_2_ − OD_0_)) × 100 (2)

where OD_0_ represents the absorbance of the negative control group (distilled water rather than the sample solution), OD_1_ represents the absorbance of the experimental group, and OD_2_ represents the absorbance of the normal group (distilled water rather than hydrogen peroxide).

#### 2.7.3. DPPH (2,2-Diphenyl-1-picrylhydrazyl) Radical-Scavenging Ability

The DPPH radical scavenging activity was measured following the methods reported by Chen et al., (2018) [27]. In an equivalent amount of 0.1 μM DPPH ethanol solution, samples of various concentration (0.2, 0.4, 0.6, 0.8, and 1.0 mg L^−1^) were added. Ascorbic acid was used as a positive control. After 30 min of dark reaction at room temperature (25 °C), the absorbance was measured at 517 nm. The following equation was used to calculate the DPPH radical-scavenging activity:

Scavenging capacity (%) = (1 − (OD_1_ − OD_2_)/ OD_0_) × 100 (3)

where OD_0_ is the absorbance of the control group (distilled water rather than the sample solution); OD_1_ is the absorbance of the experimental group; OD_2_ is the absorbance of the blank group (ethanol rather than DPPH).

#### 2.7.4. Superoxide Anion Radical-Scavenging Ability

The superoxide anion free radical test kit (Jiancheng, Nanjing, China) was used to determine the superoxide anion scavenging activity of the sample (1.0, 2.0, 3.0, 4.0, 5.0 mg L^−1^), which is based on simulating the reaction system of xanthine and xanthine oxygenase in the body. Ascorbic acid was used as a positive control. The following equation was used to calculate the DPPH radical-scavenging activity:

Scavenging capacity (%) = (1 − (OD_1_/OD_0_)) × 100 (4)

where OD_0_ is the absorbance of the control group (distilled water rather than the sample solution), OD_1_ is the absorbance of the experimental group.

### 2.8. Statistical Analysis

All assays were repeated with three independent biological replicates and three technical replicates. The SPSS 18.0 was used for one-way analysis of variance (ANOVA) with subsequent post hoc multiple-comparison LSD tests. The IC_50_ (half maximal inhibitory concentration) was determined using GraphPad Prism 7 to characterize the effective concentration of EPS when the scavenging activity of free radicals in the system reaches 50%. The results were considered significant at differences of *p* < 0.05 and represented as mean ± standard deviations error of mean.

## 3. Results

### 3.1. Morphological and Molecular Identification of Microalgal Strain

**Figure 1:**
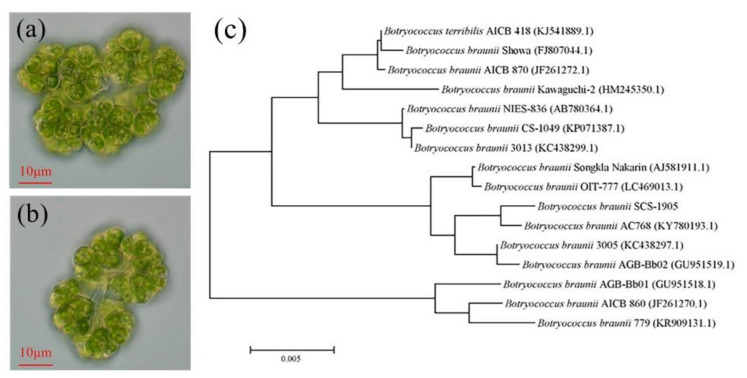
Cell morphology of *Botryococcus braunii* SCS-1905 (**a**,**b**) and the phylogenetic tree based on 18S rRNA gene sequence of *Botryococcus braunii* species using the neighbor-joining method (**c**).

The single cells were oval in shape and covered on the top of the irregularly branched, translucent glue colony, as seen under optical microscopy (×400) (Figure 1a,b). In addition, the cell had a yellow-green pigment body that is cup-shaped or leaf-shaped. With a population diameter of 40–70 μm, single cells were 3–6 μm in width and 6–12 μm in length. The morphology characteristics of strain SCS-1905 conformed to *B*. *braunii* species.

Total base pairs of 18S rRNA gene of SCS-1905 were obtained by sequencing. BLAST analysis revealed that strain was closely related to *B*. *braunii* AC768 (99.9%) (Figure 1c). Based on the morphological and molecular analyses, it was preliminarily concluded that the microalgal strain SCS-1905 belonged to Chlorophyta, Trebouxiophyceae, Trebouxiales, Botryococcaceae, and *Botryococcus braunii*.

### 3.2. Preparation and Chemical Composition of EPS

**Figure 2:**
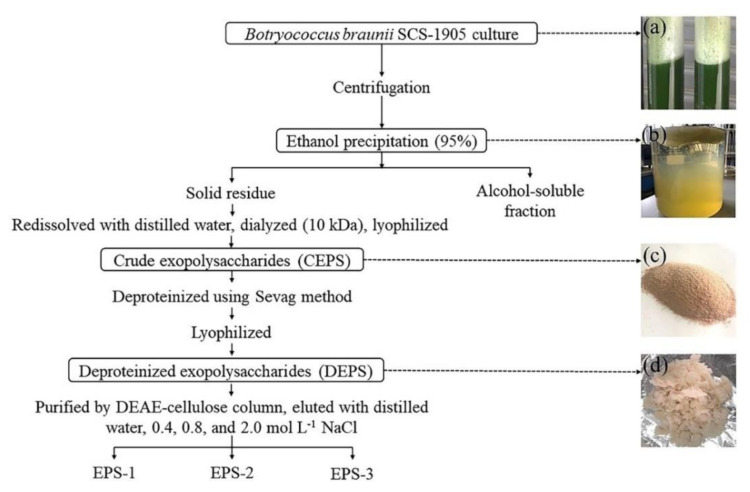
The extraction and purification procedure of exopolysaccharides. (**a**) culture, (**b**) ethanol precipitation, (**c**) crude exopolysaccharides (CEPS), and (**d**) deproteinized exopolysaccharides (DEPS).

The extraction and purification for *B*. *braunii* SCS-1905 EPS followed the procedure outlined in Figure 2. During the growth process of *B*. *braunii* SCS-1905, a large amount of white foam was investigated in the upper layer of the culture (Figure 2a). After the addition of 95% ethanol to the supernatant, large amounts of floating flocculent appeared (Figure 2b). CEPS was extracted from the culture adopting 95% ethanol precipitation, dialysis desalination, and lyophilization. DEPS was obtained after deproteinization using the Sevag method, and EPS purification was performed using the DEAE-cellulose column. The desalted and freeze-drying CEPS was a yellow powder and had no obvious odor (Figure 2c). Compared with CEPS, the flocculent DEPS was white in color and had higher solubility (Figure 2d). In the end, three kinds of novel exopolysaccharides termed EPS-1, EPS-2, and EPS-3 were isolated, with a total recovery of 40.53% after purification from DEPS.

**Figure 3:**
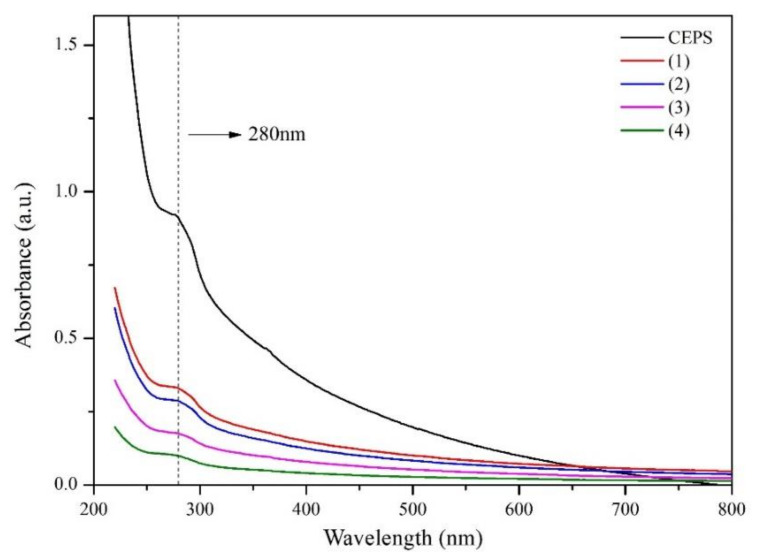
Absorption spectrum of exopolysaccharides. CEPS: crude exopolysaccharides, (1) first deproteinized procedure, (2) second deproteinized procedure, (3) third deproteinized procedure, (4) fourth deproteinized procedure.

The absorption spectrum of CEPS solutions was determined during the deproteinized process (Figure 3). The presence of aromatic amino acids with conjugated double bonds in proteins displayed a distinct corresponding UV peak with an absorption maximum at 280 nm, thus the presence of proteins in the sample can be determined by a UV-visible spectrophotometer. As deproteinization proceeded sequentially, the protein content decreased sequentially in the samples compared to CEPS. Deproteinization was repeated four times, the scanning curve was virtually flat, and the samples were collected after the fourth deproteinization, named DEPS.

The properties of CEPS and DEPS, including carbohydrate, protein, uronic acid, and SO_4_^2−^ contents, are summarized in Table 1. The preliminary chemical composition results showed that the total carbohydrate, protein, uronic acid, and SO_4_^2−^ contents of CEPS were 42.39%, 4.04%, 7.43%, and 1.95%, respectively. While the preliminary chemical composition of DEPS was significantly different from CEPS, the total carbohydrate of DEPS was increased to 58.88% DW, an increase of 38.90%, and the uronic acid content was increased to 8.83% DW, an increase of 18.84%. The sulfuric acid content was reduced to 1.52% DW, which was a reduction of 22.05%, and the protein content was reduced to 2.3% DW, which was a reduction of 43.06%.

### 3.3. Purification and Chemical Composition of Purified Fractions

**Figure 4:**
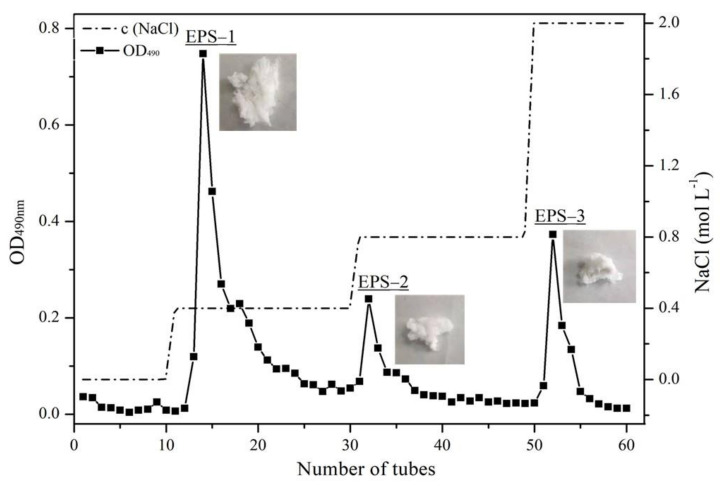
Elution profiles of exopolysaccharides by DEAE-cellulose column chromatography with gradient of NaCl solution (0, 0.4, 0.8, and 2.0 mol L^−1^).

By eluting with different concentrations of NaCl solution, three fractions were collected, namely EPS-1 (eluted with 0.4 M NaCl solution), EPS-2 (eluted with 0.8 M NaCl solution), and EPS-3 (eluted with 2.0 M NaCl solution). According to the elution peaks, the fractions were merged in order. The polysaccharide samples were obtained after ethanol precipitation, dialysis, and lyophilization, which accounted for 54.06%, 23.24%, and 22.70%, respectively (Figure 4).

Subsequently, the total carbohydrate, protein, and uronic acid content of the three purified polysaccharide fractions were determined (Table 2). The total carbohydrate and protein content of the three fractions were significantly different (*p* < 0.05). Total carbohydrate content of EPS-1, EPS-2, and EPS-3 were 53.27% DW, 42.36% DW, and 39.15% DW, respectively, and the protein content was 0.95% DW, 0.80% DW, and 0.86% DW, respectively. Uronic acid content was 7.04% DW, 6.82% DW, and 6.56% DW, respectively, with no significant difference (*p* > 0.05).

### 3.4. Primary Structures Characterization of Purified Fractions

**Figure 5:**
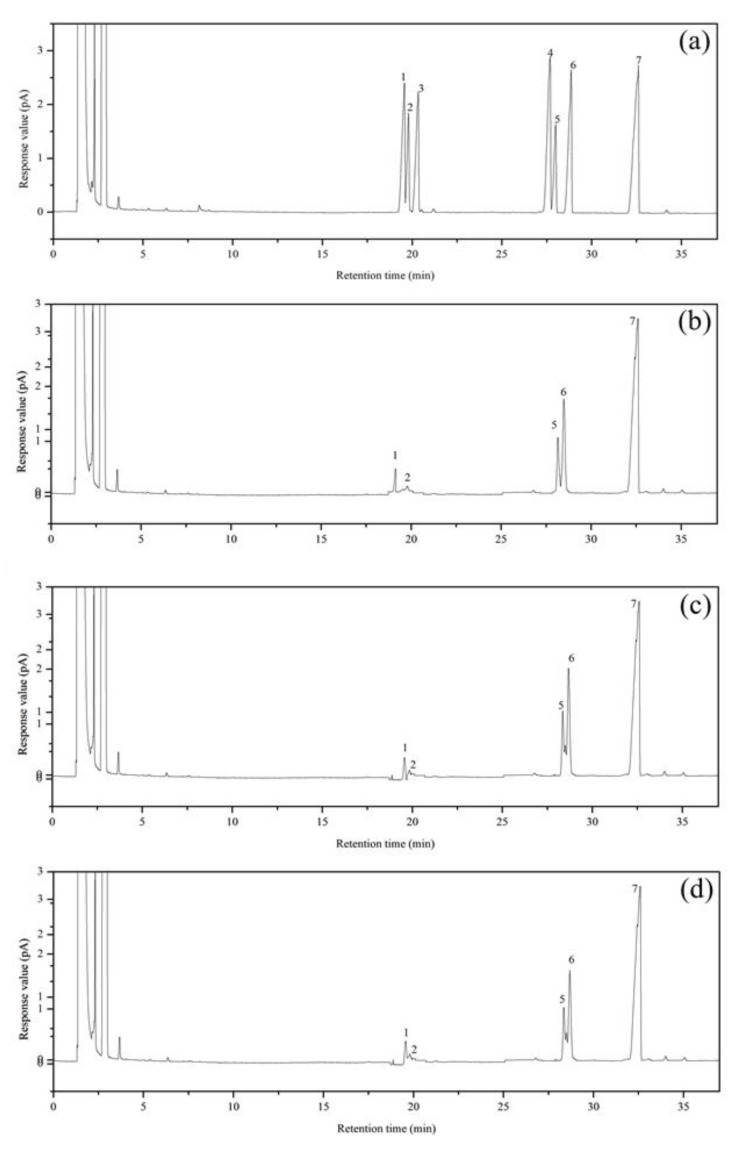
Gas chromatography profiles of (**a**) mixed standard monosaccharides, (**b**) EPS-1, (**c**) EPS-2, and (**d**) EPS-3. The peaks in chromatography profile (**a**) from left to right order are as follows: (1) arabinose; (2) fucose; (3) xylose; (4) mannose; (5) glucose; (6) galactose; (7) inositol. EPS: exopolysaccharides.

The monosaccharide compositions of EPS-1, EPS-2, and EPS-3 were determined by gas chromatography, and the results are shown in Table 3. The retention time of arabinose, fucose, xylose, mannose, glucose, and galactose was 19.58 min, 19.80 min, 20.33 min, 27.78 min, 28.80 min, and 28.90 min, respectively (Figure 5). The *B*. *braunii* SCS-1905 EPS was mainly composed of four monosaccharides, including arabinose, fucose, glucose, and galactose, among which the content of galactose is the highest (52.34–54.12%), followed by glucose (34.60–35.53%) and arabinose (9.41–10.32%); fucose content is the lowest, accounting for only about 2%. EPS-1, EPS-2, and EPS-3 showed no significant difference in the percentage content of glucose and fucose (*p* > 0.05), while EPS-3 expressed a remarkable high amount of arabinose and low galactose content (*p* < 0.05).

**Figure 6:**
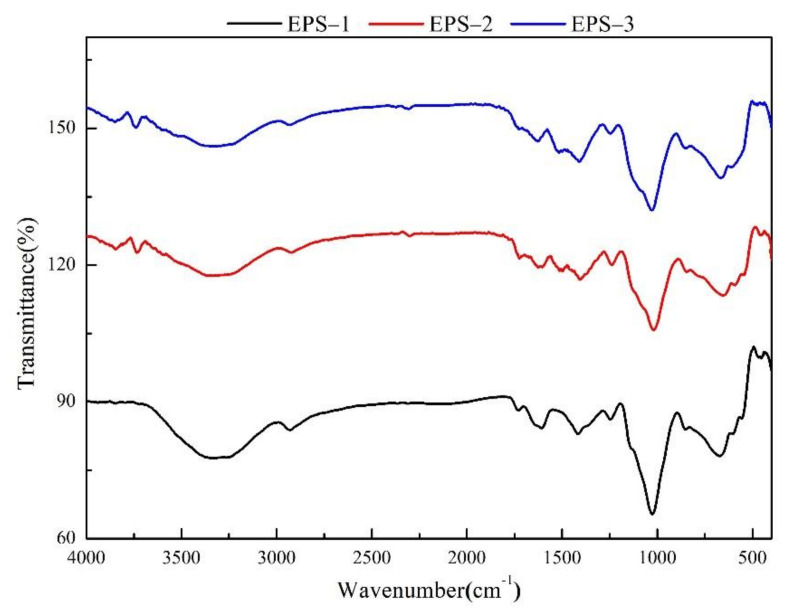
Fourier-transform infrared absorption spectra of purified fractions, EPS: exopolysaccharides.

The Fourier-transform infrared absorption (FT-IR) spectra of three purified fractions within the range of 4000–400 cm^−1^ are depicted in Figure 6. The infrared peaks were assigned according to the existing literature. The broad absorption peaks at 3326−3338 cm^−1^ were the stretching vibrations of hydroxyl (−OH); the weak absorption peaks at 2925−2931 cm^−1^ may be related to the stretching vibration of the C−H bond on the sugar ring [28]. The above two bonds are typical infrared absorption peaks of polysaccharides. The absorption peaks that appeared at 1612 cm^−1^ were derived from the stretching vibration of the −CHO and −C=O bond of the −COOH group in uronic acid [29]. Protein characteristic absorption at 1411 cm^−1^ indicated the presence of small amounts of protein [30]. The absorption peaks appearing at 1244 cm^−1^ (EPS-1), 1238 cm^−1^ (EPS-2), 1246 cm^−1^ (EPS-3), and 1023 cm^−1^ (EPS-1), 1035 cm^−1^ (EPS-2), 1026 cm^−1^ (EPS-3) corresponded to the stretching vibration of the C−O bond in C−OH and C−O−C [31]. The absorption bands at 1149 cm^−1^ (EPS-1), 1147 cm^−1^ (EPS-2), and 1155 cm^−1^ (EPS-3) represented the presence of *β*-glycosidic bonds. The absorption bands at 853 cm^−1^ (EPS-1), 856 cm^−1^ (EPS-2), and 864 cm^−1^ (EPS-3) were generally caused by the vibration of a pyranose ring with a glycosidic bond type of *β*-configuration [30]. The infrared spectroscopy results showed that EPS-1, EPS-2, and EPS-3 all have typical absorption peaks of polysaccharide, and there was a pyranose ring with a glycosidic bond type of *β*-configuration [32].

### 3.5. Evaluation of Antioxidant Activity

**Figure 7:**
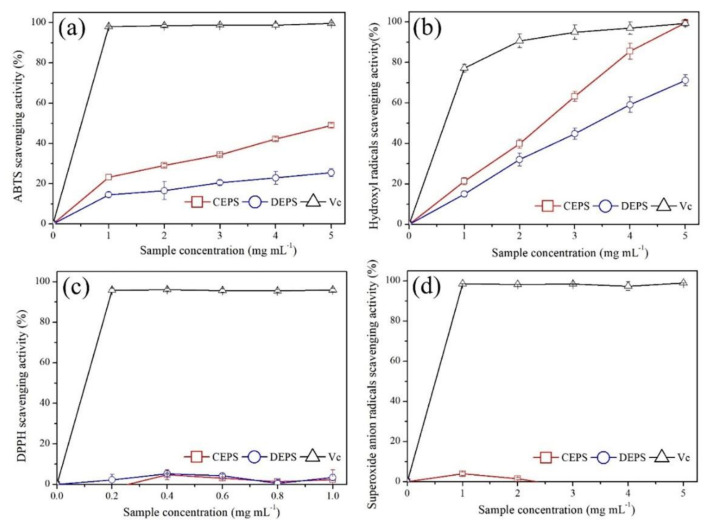
Radical-scavenging activity of crude exopolysaccharides and deproteinized exopolysaccharides. (**a**) ABTS, (**b**) hydroxyl radicals, (**c**) DPPH, and (**d**) superoxide anion radicals. CEPS: crude exopolysaccharides, DEPS: deproteinized exopolysaccharides, Vc: Ascorbic acid. (n = 3; data: mean ± SD).

The antioxidant activity of *B*. *braunii* EPS was evaluated by measuring the scavenging activity against four free radicals (ABTS, hydroxyl, Superoxide anion, and DPPH radical), and the results are shown in Figure 7. Due to the low polarity requirement of the sample, ABTS is extensively utilized as a non-physiological free radical in in vitro antioxidant assays. Figure 7a showed the scavenging activities of CEPS and DEPS against ABTS radicals. When the concentration was in the range of 1.0–5.0 mg mL^−1^, the scavenging rate of ABTS by CEPS and DEPS increased in a dose-dependent manner, and the maximum scavenging activity reached 48.93% and 25.51% when the concentration was 5.0 mg mL^−1^, respectively, with CEPS significantly higher than DEPS (*p* < 0.05). The IC_50_ of the samples against ABTS radicals were calculated using GraphPad Prism 7 software and were 5.12 mg mL^−1^ and 12.38 mg mL^−1^ for CEPS and DEPS, respectively (Table 4).

The hydroxyl radical scavenging ability of CEPS and DEPS ranging from 1.0–5.0 mg mL^−1^ concentrations are depicted in Figure 7b. The maximum scavenging activities of CEPS and DEPS were 99.50% and 71.15%, respectively, corresponding to IC_50_ of 1.67 mg mL^−1^ and 3.04 mg mL^−1^ (Table 4). The scavenging activity of CEPS was up to 99.50% at 5 mg mL^−1^, the same as ascorbic acid. Compared with ABTS free radicals, *B*. *braunii* EPS had significant hydroxyl radicals scavenging activity.

DPPH is a non-physiological free radical that is employed to assess the antioxidant capacity of active compounds that are soluble in polar reagents, such as lipids and phenols. DPPH radical scavenging activity of CEPS and DEPS with the concentrations ranging from 0.2 to 1.0 mg L^−1^ were depicted in Figure 7c. Neither CEPS nor DEPS showed concentration-dependent DPPH radical scavenging activity that concluded the two had no DPPH scavenging activity. This might be due to the fact that polysaccharides are insoluble in ethanol and the contact of polysaccharide with the DPPH reagents would produce insoluble substances and appear turbid, making the absorbance value readings more incorrect.

The superoxide anion radical scavenging activities of CEPS and DEPS were exhibited in Figure 7d. Superoxide anion radicals scavenging activities of both CEPS and DEPS did not show concentration dependence at the concentration range of 1.0–5.0 mg mL^−1^, hence both were considered to have no O_2_^-^ scavenging activity.

In conclusion, *B*. *braunii* EPS exhibits antioxidant activity against various radicals in vitro, but was not very potent, as evidenced by its lack of scavenging ability against DPPH and superoxide anion radicals. The activity of CEPS was significantly higher than DEPS, and the antioxidant capacity of both was mainly shown by the scavenging ability of hydroxyl radicals.

## 4. Discussion

Based on morphological and molecular identification, it was concluded that the microalga strain SCS-1905 belonged to Chlorophyta, Trebouxiophyceae, Trebouxiales, Botryococcaceae, *Botryococcus*, *Botryococcus braunii*, and showed a close phylogenetic relationship with *B*. *braunii* AC768. Accordingly, it was classified as race L [33]. Previous studies mostly focused on the excellent hydrocarbon production properties and were committed to its development and application as a source of biofuels, due to the excellent performance in synthesizing and accumulating various lipids and hydrocarbons [34]. Not only that, *B*. *braunii* could secrete large amounts of polysaccharides into the surroundings, with the production up to 5.0 g L^−1^ [35]. Because of its remarkable exopolysaccharide synthesis capabilities, *B*. *braunii* seems to be a promising candidate for microalgal EPS industrialization. Although some studies have investigated the factors that affect *B*. *braunii* EPS secretion and outdoor cultivation, the physicochemical and structural properties, as well as biological activity, have not been documented in detail. In this study, we isolated a new strain of *B*. *braunii* and purified the EPS, focusing on the composition and antioxidant activities in vitro.

The chemical composition and structure of *B*. *braunii* SCS-1905 EPS have been characterized. The results of infrared spectroscopy (1411 cm^−1^) and protein content determination by the Lowry method (2.3% DW) confirmed that proteins were still present in the polysaccharides after the Sevage method treatment, implying that the exopolysaccharide of *B*. *braunii* may be a proteoglycan [36,37]. Allard and Casadevall (1990) reported the existence of proteins in EPS of race A, B, and L in the range of 5–10% DW, which is consistent with our findings [17]. To date, the structural analysis of *B*. *braunii* EPS has not been extensively studied, and chemical methods alone are not enough to determine that it is a proteoglycan. The first structural characterization of race B EPS was recently carried out, demonstrating that the *B*. *braunii* polysaccharide is composed of a 3-linked α-Gal*p* backbone, with 4-O-methyl-D-glucopyranuronic acid and D-galactopyranose (6-deoxyaltropyranose and 6-deoxyaltrofuranose as terminal residues) [38]. Nevertheless, the monosaccharide composition and ratios of the four races of *B*. *braunii* vary greatly among each other, and therefore are not universal and do not provide a generalized and comprehensive description of the structural features In addition to excreting polysaccharides to the surrounding environment, the polysaccharide fibrillar sheath in the cell wall is also one of the sources of EPS [15]. Since the daughter cells of *B*. *braunii* will detach from the mother cell to form a new colony, the polysaccharide sheath of the mother cell will fall off and free in the medium during this process. Tatli et al., (2018) analyzed the morphology and structural components of the complex extracellular matrix of *B*. *braunii* and found that the colony was coated with arabinose (46.7%) and galactose (36.3%) [39]. In the formed polysaccharide fibrillar sheath, the protein content accounted for about 1.25%, which might be connected to the fibrillar formation. Subsequent studies showed that the polysaccharide associated protein was a unique extracellular matrix hydroxyproline-rich glycoprotein that involved ECM polysaccharide fiber biosynthesis [40]. Since the composition of the polysaccharide sheath shed from the top of the cell during the process of cell division had merely been determined, it was impossible to confirm whether the *B*. *braunii* EPS was exclusively composed of the proteoglycan.

The purified polysaccharide fractions EPS-1, EPS-2, and EPS-3 were obtained by elution with different concentrations of NaCl solution, and the monosaccharide composition and infrared spectra showed no significant difference. The results of infrared spectroscopy showed that *B*. *braunii* SCS-1905 EPS had typical absorption peaks of polysaccharides and contained uronic acid. Due to the excellent ability to chelate metal ions, polysaccharides containing uronic acid groups could be useful as sewage treatment agents and heavy metal antidotes [41,42]. *B*. *braunii* SCS-1905 EPS was mainly composed of galactose, glucose, arabinose and a small amount of fucose. Our results were consistent with previous studies. Metzger and Largeau (2005) analyzed the exopolysaccharides of 16 different strains of *B*. *braunii* and found that galactose was the primary monosaccharide in 10 of them, while glucose was the main monosaccharide in the other six [33]. Allard and Casadevall (1990) also identified several uncommon O-methylated sugars, including 3-O-methylrhamnose, 3-O-methylfucose and 6-O-methylhexose [17]. Galactose, the main scaffold present in the exopolysaccharide fraction, is an interesting monosaccharide for the chemical industry as bioethanol and for the food industry as a prebiotic [43,44]. Moreover, *B*. *braunii* EPS could be a source of fucose, a 6-deoxy sugar that was a valuable substrate in the chemical production of flavoring compounds [35].

Microalgal EPS are widely acknowledged for a variety of excellent biological activities, such as antioxidant, anti-tumor, antibacterial and immune activity, while the biological activity of *B*. *braunii* EPS has not been reported [45,46,47,48]. The various biological activities of natural polysaccharides are the result of a complex interaction of diverse structural features, including molecular weight, monosaccharide composition, degree of sulfation and sulfation position, protein content and stereochemical features [48]. For instance, the anticoagulant activity of fuciodan is determined by a combination of their monosaccharide composition, molecular weight, degree of sulfation and sulfation position. Harden et al., (2009) demonstrated that galactans, fucans and galactofucans with different structures, sulfation patterns, and molecular weights all inhibited herpes simplex virus type I and II (HSV) [49]. In contrast, Fernandez et al., (2010) found that the degree of branching of polysaccharides significantly increased their biological activity and that branched fucoidan oligosaccharides had higher anti-inflammatory activity than straight-chain oligosaccharides [50]. The structure–activity relationships of microalgal EPS have not been adequately studied. In this study, the scavenging activities of *B*. *braunii* EPS against four free radicals in vitro were evaluated to provide references for the subsequent development and utilization. The strain *B*. *braunii* SCS-1905 isolated in this study showed strong hydroxyl radical scavenging activity (IC_50_ = 1.67 mg mL^−1^). Hydroxyl radicals possessed the strongest oxidation ability among the reactive oxygen radicals, resulting in excessive carbohydrate peroxidation, amino acids, proteins, nucleic acids, and other molecules [25]. Previous studies showed that the carboxyl group in the uronic acid group could reduce the formation of hydroxyl radicals by chelating metal ions [51]. Therefore, the strong antioxidant activity of *B*. *braunii* SCS-1905 EPS might be due to the uronic acid group chelating Fe^2+^ to reduce the generation of hydroxyl free radicals. However, the same strong antioxidant capacity was not observed in the scavenging assay of ABTS. The EPS had relatively poor scavenging capacity for ABTS, and the scavenging activity was only 25.51% when the polysaccharide concentration was 5.0 mg mL^−1^. Despite the fact that the ABTS assay employed non-physiological free radicals, it may indicate whether a compound may directly scavenge free radicals [52]. The elimination of ABTS free radicals was caused by the electron transfer reaction of antioxidants, and sulfate acid groups were generally used as electron donors in polysaccharides [53]. The results of Liu et al., (2016) on antioxidant activity of *Flammulina velutipes* polysaccharides, suggesting higher acidic polysaccharide contents led to stronger ABTS scavenging activity [54]. In previous studies, no sulfate groups were found in *B*. *braunii* EPS, while in this study, *B*. *braunii* SCS-1905 EPS was detected with trace sulfate content (less than 2% DW), so the weak scavenging activity of EPS against ABTS from *B*. *braunii* might be caused by its low sulfate content [17,55]. The study into the antioxidant activity of sulfated polysaccharide fractions from *Porphyra haitanesis* has demonstrated that the sulfate content was not the only factor that influences the antioxidant activity, which is also affected by protein content. The higher the protein content, the stronger the antioxidant activity [56].

## 5. Conclusions

The newly isolated *Botryococcus braunii* SCS-1905 was identified as belonging to the chemical race L of *B*. *braunii*, with the potential to produce EPS. *B*. *braunii* SCS-1905 EPS were macromolecular polysaccharides containing uronic acid, protein and a low content of sulfuric acid groups. The monosaccharide composition included galactose, glucose, arabinose and fucose, and there were pyranose rings with a glycosidic bond type of *β*-configuration. Moreover, *B*. *braunii* SCS-1905 EPS indicated a considerable hydroxyl radical scavenging activity. Therefore, *B*. *braunii* could be regarded as a promising alternative strain of microalgal EPS applied in the pharmaceutical, nutraceutical, and cosmeceutical industries as an alternative polysaccharide source.

## Figures and Tables

**Table 1:** Chemical composition of exopolysaccharides.

Chemical Composition (%DW)	Sample	
	CEPS	DEPS
Total carbohydrate	42.39 ± 0.15	58.88 ± 1.23
Protein	4.04 ± 0.14	2.30 ± 0.08
Uronic acid	7.43 ± 0.47	8.83 ± 0.99
Sulfate	1.95 ± 0.01	1.52 ± 0.04

DW: dry weight, CEPS: crude exopolysaccharides, DEPS: deproteinized exopolysaccharides (*n* = 3; data: mean ± SD).

**Table 2:** Chemical composition of purified polysaccharide fractions.

Chemical Composition (%DW)	Sample		
	EPS-1	EPS-2	EPS-3
Total carbohydrate	53.27 ± 0.09 ^a1^	42.36 ± 0.18 ^b1^	39.15 ± 0.17 ^c1^
Protein	0.95 ± 0.01 ^a2^	0.80 ± 0.02 ^c2^	0.86 ± 0.01 ^b2^
Uronic acid	7.04 ± 0.50 ^a3^	6.82 ± 0.42 ^a3^	6.56 ± 0.39 ^a3^

DW: dry weight, EPS: exopolysaccharides. Different letters denoted significant differences among the percentage of different chemical components of EPS-1, EPS-2, and EPS-3 (^a1–c1^: Total carbohydrate; ^a2–c2^: Protein; ^a3^: Uronic acid).

**Table 3:** Chemical composition of exopolysaccharides.

Percentage (%Total Carbohydrate)	Sample		
	EPS-1	EPS-2	EPS-3
Galactose	54.12 ± 0.19 ^a1^	53.82 ± 0.80 ^a1^	52.34 ± 0.61 ^b1^
Glucose	34.60 ± 0.02	34.65 ± 0.86	35.53 ± 0.20
Arabinose	9.41 ± 0.09 ^b2^	9.54 ± 0.24 ^b2^	10.32 ± 0.16 ^a2^
Fucose	1.87 ± 0.25	1.99 ± 0.18	1.80 ± 0.25

DW: dry weight, EPS: exopolysaccharides. Different letters denoted significant differences among the percentage of different monosaccharide composition of EPS-1, EPS-2, and EPS-3 (^a1–b1^: galactose; ^a2–b2^: arabinose).

**Table 4:** IC_50_ of crude exopolysaccharides and deproteinized exopolysaccharides.

IC_50_ (mg·mL^−1^)	Sample		
	CEPS	DEPS	Vc
ABTS	5.13 ± 0.11 ^b1^	12.38 ± 1.06 ^a1^	0.003 ± 0.001 ^c1^
OH·	1.67 ± 0.06 ^b2^	3.04 ± 0.30 ^a2^	0.240 ± 0.001 ^c2^

DW: dry weight, CEPS: crude exopolysaccharides, DEPS: deproteinized exopolysaccharides, Vc: Ascorbic acid, ABTS: 2, 2′-azinobis (3-ethylbenzothiazoline-6-sulfonic acid, OH: hydroxyl radical, IC_50_: half maximal inhibitory concentration. Different letters denote significant differences among the values of IC_50_ of different free radicals scavenging activity of CEPS and DEPS (^a1–c1^: ABTS; ^a2–c2^: OH·).

## Data Availability

No new data were created or analyzed in this study. Data sharing is not applicable to this article.
